# Aspects of the pathology induced by *Spinitectus petterae* Boomker, 1993 in the stomach of *Clarias gariepinus* (Burchell, 1822) using light and scanning electron microscopy

**DOI:** 10.1111/jfd.13611

**Published:** 2022-04-14

**Authors:** Lucinda Austin, Quinton Marco Dos Santos, Annemariè Avenant‐Oldewage

**Affiliations:** ^1^ 61799 Department of Zoology University of Johannesburg Johannesburg South Africa

**Keywords:** African sharptooth catfish, Clariidae, endoparasites, helminth, South Africa

## Abstract

*Spinitectus* spp. (Rhabdochonidae) are enteric nematodes characterized by annular spines. At the anterior end, these spines assist attachment and aid penetration into the host tissue. During parasitological surveys of the Vaal River system from three localities, below the Vaal River Barrage in the Vaal Dam surrounding UJ island and below the Grootdraai Dam, *Spinitectus* specimens were collected from the stomach lining of the sharptooth catfish, *Clarias gariepinus* (Burchell, 1822). Histopathological effects induced by *Spinitectus petterae* Boomker, 1993 on *C. gariepinus* has not been studied. Thus, the aim of this study was to describe the histopathology induced by *S. petterae*. For light microscopic examination, tissue samples with attached *S. petterae* were sectioned and stained with haematoxylin and eosin. Additionally, attached nematodes were also studied using scanning electron microscopy. Leukocytes were counted with the Disector principle. Standard infection parameters (prevalence, mean intensity and abundance) were calculated and compared to host parameters. Prevalence varied greatly (11.77% to 100%) between localities. Histopathology induced by *S. petterae* to *C. gariepinus* stomach (cardiac region) consisted of significant leukocyte infiltration, acute ulcerations and chronic granuloma formation. This was similar to the pathology of other *Spinitectus* occurring in host anterior intestine and stomach, but granuloma formation had not been previously reported and this suggests chronic infection in wild caught fish.

## 1 INTRODUCTION

*Spinitectus* are medium‐sized nematodes that infect the digestive tract of marine and freshwater fishes, as well as amphibians (Anderson, 2000, 2009). They are characterized by distinct transverse annular cuticular rings (Anderson, 2000, 2009). The anterior bears blunt and broad pseudo labia (Moravec, 2019) and a weakly sclerotized stoma (Moravec, 2019). The oesophagus consists of an anterior muscular and posterior multinucleated glandular section (Boomker, 1993; Boomker & Puylaert, 1994). The annular spines are used to penetrate the mucosa and aid in movement and attachment within the host's stomach, intestine and mesenteries (Jilek & Crites, 1982a; Meguid & Eure, 1996). Movement through the gastrointestinal lumen can cause disruption when *Spinitectus* wriggle between villi (Jilek & Crites, 1982a; Meguid & Eure, 1996).

The effect of *Spinitectus* spp. on fishes has only been reported for four species, all from the Americas (summarized in Table 1). *Spinitectus carolini* Holl, 1928 penetrated the mucosa and submucosa in the anterior intestine of both the green sunfish, *Lepomis cyanellus* Rafinesque, 1819, and the bluegill sunfish, *Lepomis macrochirus* Rafinesque, 1819, from North America (Jilek & Crites, 1982a; Meguid & Eure, 1996). *Spinitectus jamundensis* Thatcher and Padilha, 1977, however, penetrates the mucosa in the cardiac and pyloric region of the stomach of streaked prochilod (*Prochilodus lineatus* (Valenciennes, 1836)) from South America (Ramallo et al., 2000). Laboratory induced infection with 3rd stage larvae of *Spinitectus micracanthus* Christian, 1972 moved freely, causing no damage or host reaction (Keppner, 1975), similar to *S. carolini* larvae during early infection (Jilek & Crites, 1982a). Larvae of *S. micracanthus* attaches to the anterior intestine, pyloric caeca and rectum, but no pathological effect was observed during the adult stage (Meguid & Eure, 1996). In heavy infections, *S. carolini* burrows through both the mucosa and submucosa of *L. cyanellus* and *L. macrochirus*, causing severe damage to the tissue (Jilek & Crites, 1982a; Meguid & Eure, 1996). An extensive build‐up of yellow fluid, containing antibodies, was reported in the anterior intestine in both studies (Jilek & Crites, 1982a; Meguid & Eure, 1996). The increase in mucus was due to significant hyperplasia of goblet cells (*t* = 3.83; *p* < .05) in the anterior intestine (Meguid & Eure, 1996). Furthermore, Meguid and Eure (1996) suggested that the increased mucus production is a host species‐specific reaction not caused by all *Spinitectus* species in all hosts.

**TABLE 1:** A summary of the pathological studies on *Spinitectus* species and their respective host species

Species	Host	Location	Organ system or organ	Pathology	References
*Spinitectus carolini*	*Lepomis cyanellus*	North America, North Carolina, Belew's Lake and Charlie's Pond	Anterior intestine	Accumulation of yellow mucus in stomach. Embedded in mucosa and submucosa. Gravid females penetrated to coelom, no peritonitis. Tunnels through villi. Epithelial cells damaged. Loss of columnar epithelial cells, inflammation. Connective tissue underneath mucosal layer. Ulcers; fibrotic fibre bundles in muscle layer. Goblet cell hyperplasia	Meguid and Eure (1996)
	*Lepomis macrochirus*	North America, Ohio, Laboratory	Anterior intestine	3rd, 4th stage larvae and adults; penetrated mucosa to the lamina propria. Aseptic traumatic enteritis. Damage to mucosa. Simple infectious enteritis of submucosa. Inflammation. Infiltration of eosinophils. Mucosa disrupted. Haemorrhage. Lesions. Regressive‐progressive modifications.	Jilek and Crites (1982a)
			Mesenteries	3rd, 4th stage larvae, and adults; penetrated to coelom, encapsulated in mesenteries. Fat deposits; deposits replaced by fibroblasts. Infiltration of ‘granule cells’ (sic), leucocytes, polymorphonuclear leucocytes and macrophages. Long term; necrotic fibroblasts forming fibrocystic layer, lesions	
*Spinitectus gracilis*	*Lepomis cyanellus*	North America, Ohio, Laboratory	Intestine, pyloric cecae, and mesenteries	Embedded in mucosa and submucosa. 3rd stage larvae, and adults; penetrated to coelom, encapsulated in mesenteries	Jilek and Crites (1982b)
*Spinitectus jamundensis*	*Prochilodus lineatus*	South America, Argentina, Santiago del Estero, Termas de Río Hondo	Stomach; pyloric and cardiac regions	Embedded to mucosa in pyloric region. Embedded to muscular layer in cardiac region. Lesions. Infiltration of lymphocytes	Ramallo et al. (2000)
*Spinitectus micracanthus*	*Lepomis macrochirus*	North America, Missouri, Laboratory	Pyloric cecae and rectum	3rd stage larvae; no host reaction. Adults attached to mucosa	Keppner (1975)
*Spinitectus* sp.	*Lepomis macrochirus*	–	Intestine	Embedded in mucosa. Inflammation	Hoffman (1975)
			Gills	Eggs in gill lamellae (moribund fish)	

Note

Location and organ or organ system are also listed.

During parasitological surveys in the Vaal River system (Orange River Catchment), South Africa, *Spinitectus* nematodes were collected from *C. gariepinus*. The nematodes resembled *S*. *petterae* Boomker, 1993 and were collected from the type host for this species, but from a different river system. The effects of African *Spinitectus* species on their hosts has not been described. Thus, the aim of the current study was to report the effect of *S*. *petterae* on the commercially important African sharptooth catfish, *C. gariepinus*.

## 2 MATERIALS & METHODS

### 2.1 Sample collection

**FIGURE 1:**
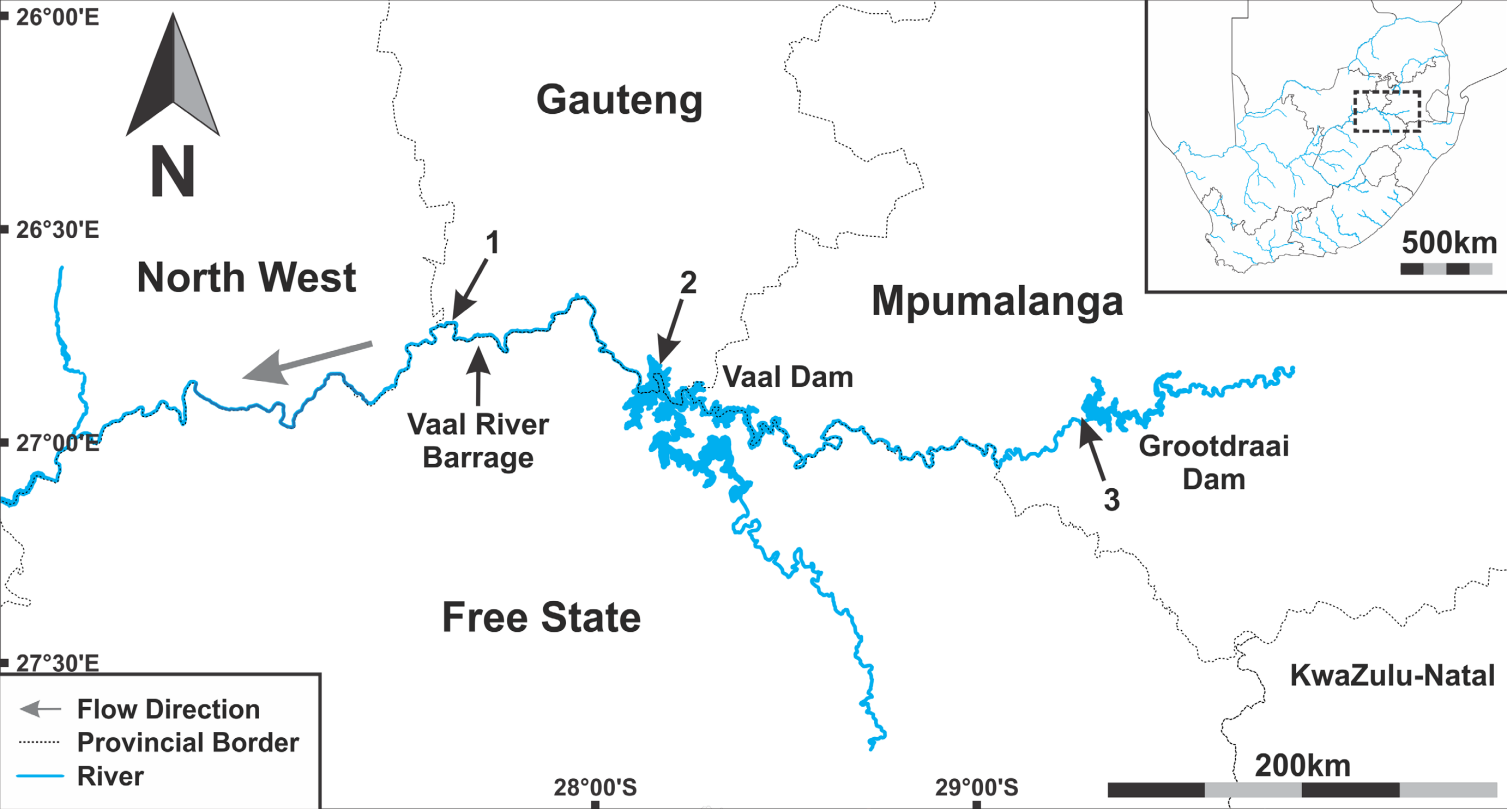
Maps of sampling localities for *Clarias gariepinus* and *Spinitectus petterae*, insert South Africa with major rivers. Sampling sites; 1, below Vaal River Barrage; 2, Vaal Dam; 3, below Grootdraai Dam

After acquiring permits from the Gauteng Department of Agriculture and Rural Development (Permit numbers: CPE2‐000125, CPE2‐0125, CPE2‐0126, CPE2‐0127), angling permits and consent from the Ethics Committee of the University of Johannesburg's Faculty of Science (2019‐04‐15/Avenant‐Oldewage_Austin), a total of 55 *C. gariepinus* were collected from three sites in the Vaal River system during February 2018 to March 2019 (see Figure 1): 1. below the Vaal River Barrage at Yellow Fish Paradise (S 26°43′50.84″, E 27°37′51.99″), 2. in the Vaal Dam, surrounding UJ island, near Deneysville (S 26°51′13.80″, E 28°8′30″), and 3. below the Grootdraai Dam near Standerton (S 26°55′22″, E 29°17′11″). During March 2018, one *C. gariepinus* was collected from the Vaal Dam near Deneysville.

Fish were collected with the use of gill nets and rod and reel. Live fish were kept in shaded, and aerated live‐wells until death. Lengths (cm) and weight (g) of each fish were recorded and thereafter, they were killed by severing the spinal cord according to the standard protocols set by the South African National Animal Ethics Guidelines. The intestine of each fish, from the oesophagus to the rectum, was removed, transferred to 0.9% saline, opened with two #5 forceps and assessed on site with brightfield and darkfield illumination for the presence of nematodes and other intestinal parasites using, a Zeiss DV4 Stereomicroscope (Carl Zeiss). Infections were recorded and tissue from the cardiac and pyloric regions with attached nematodes were removed and fixed in 10% neutral buffered formalin (NBF).

For scanning electron microscopy (SEM), ten tissue samples from four different fish (4 from the Vaal Dam and 6 from below the Grootdraai Dam) were washed under a gentle stream of tap water for 24 h, dehydrated in a stepwise increasing concentrations (30%‐100%) of ethanol followed by increasing concentrations of hexamethyldisilazane (Merck) in absolute ethanol (30%‐100%) (Nation, 1983). They were then transferred to a Sanpla Dry Keeper desiccator cabinet (Kita‐Ku, Osaka, Japan) for at least a week until completely dry. Samples were sputter coated with gold using an Emscope SC500 sputter coater (Quorum Technologies) and photomicrographs obtained using a VEGA 3 LMH scanning electron microscope (Tescan), set at 3–6 kV.

For histopathological examination, 7 NBF fixed tissue samples from 4 different fish (3 from Vaal Dam and 4 from below the Grootdraai Dam) were prepared by rinsing under a gentle stream of tap water for 24 h. Samples were dehydrated in a gradual series of ethanol to 70% ethanol in water, then further dehydrated in a gradual series of acetone (from 70% to 100%) in water. Samples were infiltrated with resin (TAAB^®^ Laboratories Ltd) under vacuum and cured at 60°C. Blocks were sectioned using a manual rotary microtome (Anglia Scientific) at 5–7 µm with a glass knife. The resin was removed with saturated NaOH in acetone (King, 1983), stained with haematoxylin and eosin and mounted with Entellan^®^ (Merck).

Photomicrographs were obtained with the aid of a Zeiss Axioplan 2 Imaging Light Microscope (Carl Zeiss) with Axiovision 4.7.2 software (Carl Zeiss). To characterize inflammation, leukocytes were counted according to the Disector principle (Kaplan et al., 2012), at the gastric gland and lamina muscularis mucosa regions. Leukocyte counts were taken from close to the infection site and at a 1150‐µm distance from infection site.

Abundance, mean intensity and prevalence were calculated as set out by Bush et al. (1997).

## 3 RESULTS

### 3.1 Light and scanning electron microscopy

**FIGURE 2:**
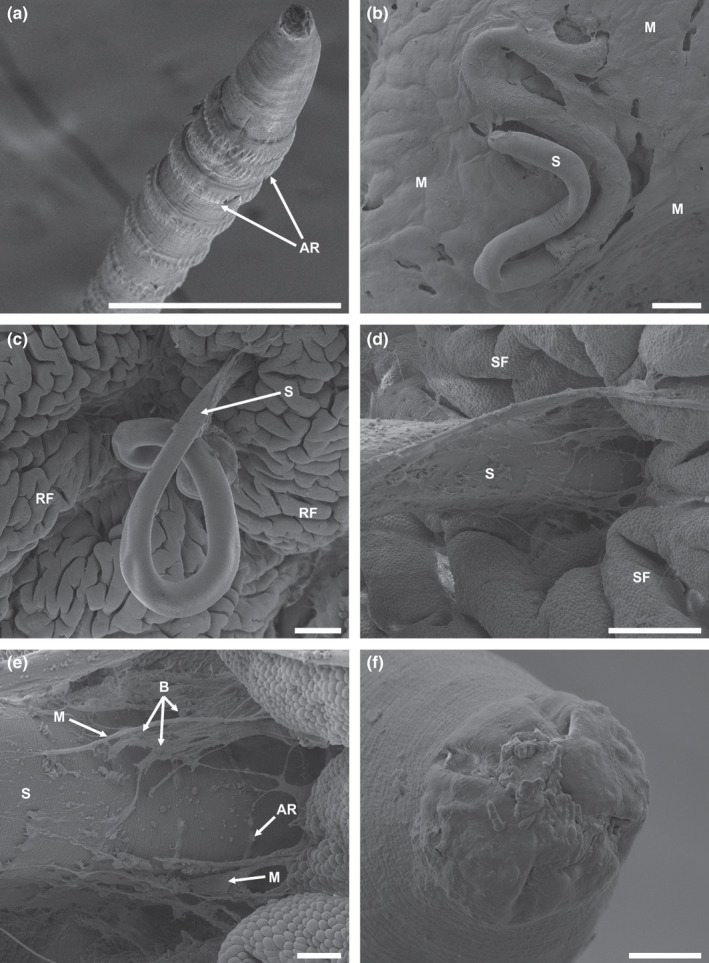
Scanning electron micrographs of *S*. *petterae* cephalic area, anterior and attachment to the stomach cardiac region of *Clarias gariepinus*. (a) Anterior of adult female, scale bar 200 µm; (b) female covered by mucus, scale bar 200 µm; (c) male with mucus removed, scale bar 200 µm; (d) male embedded into folds, scale bar 100 µm; (e) male covered by mucus strands and blebs, scale bar 20 µm; (f) larvae with possible tissue fragments and bacteria covering cephalic structures, scale bar 10 µm. AR, annular ring; B, blebs; M, mucus; RF, rugae folds; S, *Spinitectus petterae*; SF, secondary folds

*Spinitectus petterae* with characteristic annular rings (Figure 2a) occurred in the stomach's cardiac and pyloric regions of *C. gariepinus* collected from several localities in the Vaal River system. Specimens in the pyloric region appeared loosely attached to the mucosa. When samples from this region were collected, nematodes were dislodged and the attachment sites could not be macroscopically identified and were, therefore, not assessed. However, specimens in the cardiac region were deeply attached and remained so during processing. Specimens were covered by mucus (Figure 2b); however, excessive mucus was not seen, and no distinct colour change was observed. After mucus removal (Figure 2c), nematodes were observed to be attached in deep pits in the stomach rugae (Figure 2c,d). No haemorrhage or obvious inflammation of the attachment sites were observed. *Spinitectus* uses telescopic tunnelling movements to penetrate and attach to the tissue, with the remainder of the body undulating in the stomach lumen. Mucus strands that were attached to *S. petterae* contained numerous blebs and leukocytes (Figure 2d,e), and is most likely due to localized exudate, were the nematodes were attached with their anterior regions embedded up to the fourth annular ring (10%–15% of total body length). The cephalic structures of dislodged nematodes were covered by cellular material and bacteria (Figure 2f).

**FIGURE 3:**
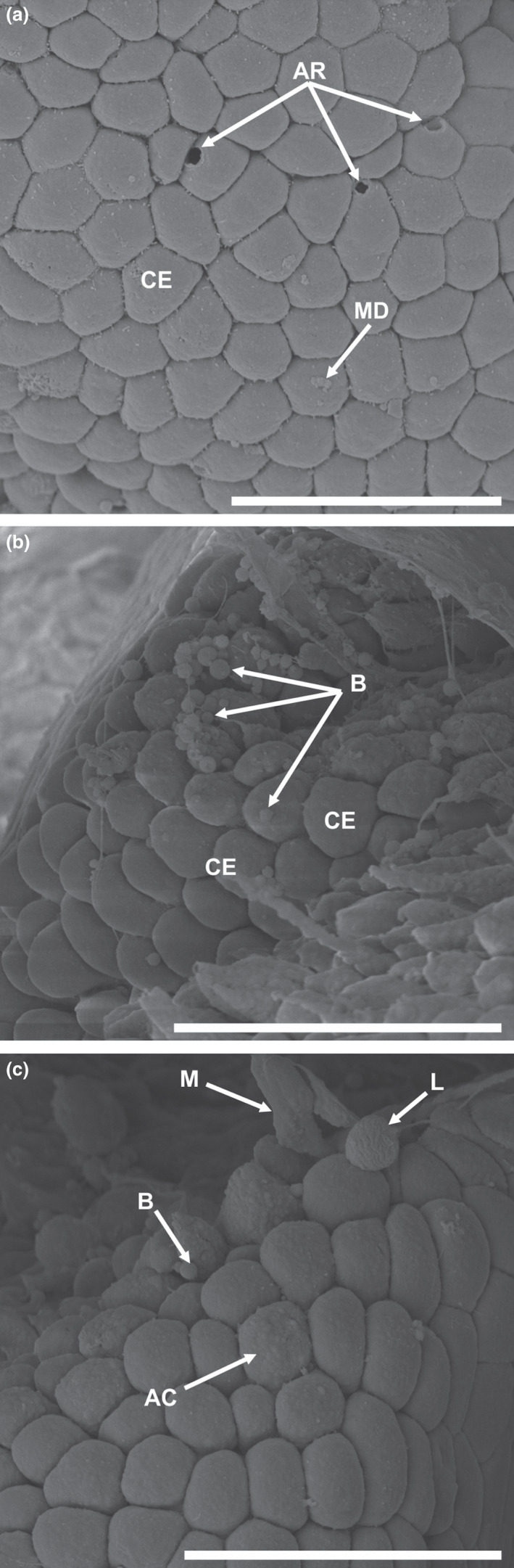
Scanning electron micrographs of the cardiac region of the stomach of *Clarias gariepinus,* uninfected (a) and tissue infected by *Spinitectus petterae* (b and c). (a) Normal columnar epithelial cells with mucin droplets and goblet cell pores, scale bar 20 µm; (b) tissue infected by *S. petterae*, showing uneven superficial texture and blebs, scale bar 20 µm; (c) tissue infected by *S. petterae* with small amounts of mucus, leukocyte‐like cells, blebs and affected columnar epithelial, scale bar 20 µm. AC, affected columnar epithelial B, bleb; CE, columnar epithelial; GC, goblet cell pore; L, leukocyte‐like cells; M, mucus; MD, mucin droplets

Columnar epithelium on the secondary folds of the normal stomach tissue structure is ordered in a flat polygon, cobblestone‐like pattern, interspersed by goblet cell pores and superficial mucin droplets (Figure 3a). Tissue impacted by *S. petterae* displayed numerous leukocytes and thrombocytes within mucus strands, alongside disrupted columnar epithelial cells (Figure 3b,c). Blebs were present on the disrupted columnar epithelium (Figure 3c). Affected cells lost their structure and were indistinctly polygon shaped with uneven superficial texture (Figure 3b,c).

**FIGURE 4:**
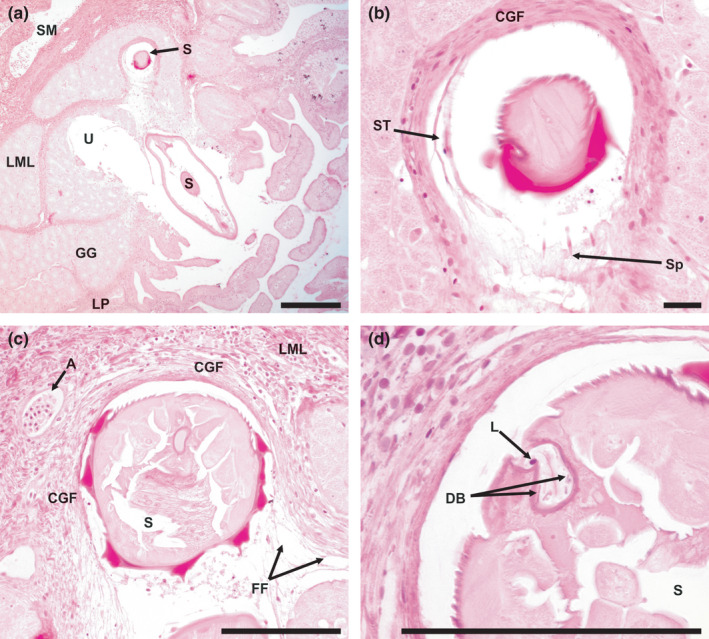
Cardiac region of *Clarias gariepinus* stomach with *S*. *petterae* attached, and a granuloma, stained with haematoxylin and eosin. (a) *S. petterae* anterior, sectioned at both the vestibule and the oesophageal regions, and an ulcer in mucosa and submucosa, scale bar 200 µm; (b) annular spines inserted into the granuloma tissue and sloughed tissue, scale bar 20 µm; (c) anterior of *S. petterae* within granuloma and infiltration of leukocytes in lamina muscularis mucosa, scale bar 100 µm; (d) section through vestibule of *S. petterae* showing a lymphocyte and diplobacillus bacteria, scale bar 100 µm. A, artery; CGF, chronic granuloma formation; DB, diplobacillus bacteria; GG, gastric glands; FF, fibrous fibres; L, leukocytes; LML, lamina muscularis mucosa; LP, lamina propria; S, *Spinitectus petterae*; SM, submucosa; Sp, spines; ST, sloughed granuloma tissue, U, ulcer

From light microscopy examination, it was clear that *S. petterae* was embedded deep in the tissue. In acute infections, tissue sloughed off by tunnelling of the nematode through the gastric glands and lamina muscularis resulted in ulcerations without bleeding (Figure 4a). These ulcerations are, furthermore, characterized by dislodged gastric glands (Figure 4b,c). In chronic infections, foreign body granulomas formed, with a circular arrangement of fibrous tissue and epithelioid cells with elongated nuclei (Figure 4b,c).

Disrupted gastric glands, adjacent to granulomas, exhibited liquefaction necrosis (Figure 4c,d). The tissue on the inside of the granuloma was disrupted by the annular spines which projected into the tissue (Figure 4b). Surrounding the granuloma, leukocyte infiltration (basophil, eosinophil and lymphocytes) and thrombocytes were present (Figure 4c,d).

The granuloma extends from the superficial epithelial layer, through the gastric layer, to the lamina muscularis mucosa layer (Figure 4a). At the apex of the granuloma, the gastric gland layer is completely destroyed and no longer separates the granuloma and lamina muscularis (Figure 4c). The granuloma is more compact or thinner at this point. Inflammation occurred in the lamina muscularis mucosa layer, but no bleeding occurred. (Figure 4a,b).

No host tissue was observed in the vestibule of *S. petterae*, but leukocytes were intermittently present and diplobacillus bacteria were consistently present (Figure 4d), there was no host tissue in the oesophagus. Deeply attached specimens displayed a medium brown intestine, unattached specimens were mostly transparent.

**FIGURE 5:**
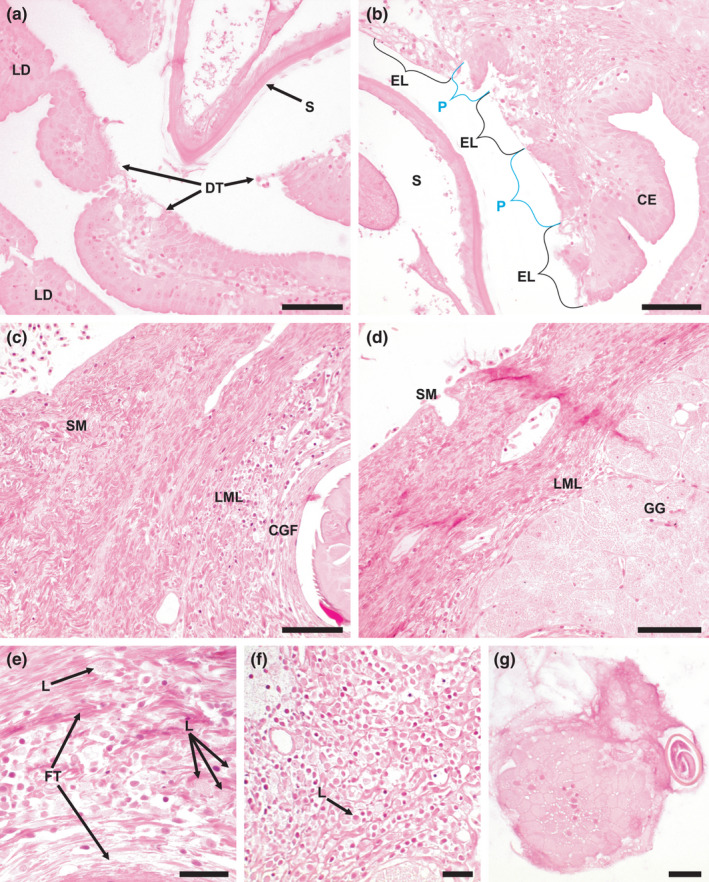
*Clarias gariepinus* cardiac stomach tissue adjacent to *S. petterae*, and unaffected and areas. (a) Effect of parasite is focal, with disrupted tissue surrounding *S. petterae*, while distant tissue shows limited or no damage, scale bar 50 µm; (b) epithelial loss and pyknosis of epithelium, scale bar 50 µm.; (c) affected tissue near granuloma, scale bar 50 µm; (d) unaffected tissue, scale bar 50 µm; (e) impacted tissue adjacent to granuloma, scale bar 20 µm; (f) unaffected lamina muscularis mucosa, scale bar 20 µm; (g) nematode egg entrapped in mucus, scale bar 20 µm. CE, columnar epithelium; CGF, chronic granuloma formation; DT, disrupted tissue; CE, columnar epithelium, EL, epithelium loss; FT, fibrous tissue; GG, gastric glands; L, leukocytes; LD, limited damage; LML, lamina muscularis mucosa; P, pyknosis; S, *Spinitectus petterae*; SM, submucosa

Continuity of the epithelial layer of the mucosa appeared disrupted by direct contact with the distal section of *S. petterae*, with some cells sloughed from the lamina propria and gastric glands (Figure 5a,b). The remaining epithelial cells underwent pyknosis (Figure 4b). Damage was localized to the attachment site (Figure 5a). Ulcers and granulomas, associated with different *S. petterae* individuals, occurred interspersed with one another in the cardiac region of the stomach.

Comparing infected (Figure 5c,e) and uninfected tissues (Figure 5d,f), infected tissue was observed adjacent to the infection site (granuloma) displaying large numbers of leukocytes. Larvated nematode eggs, trapped by mucus, occur in the lumen of the stomach (Figure 5g), these are present close to the *S. petterae* females. Measurements of these eggs are similar to the eggs within the gravid *S. petterae* females.

More leukocytes were recorded in infected (at infection site) than in uninfected tissue (far from infected tissue), while neutrophils and monocytes displayed the most significant increase at the infiltration site. This trend was followed by lymphocytes and eosinophils. Basophil counts increased insignificantly at 90% confidence levels in infected tissue. As such, all leukocyte counts differed significantly between uninfected and infected tissue, except for basophils (Table 2).

**TABLE 2:** Analysis of leukocyte numbers in the lamina muscularis mucosa, comparing normal tissue and tissue infected by *Spinitectus petterae*

*n* = 10	Range		Mean *SD*		*p*‐Value
	Normal	Infected	Normal	Infected	
Basophils	1–7	1–7	3.5; 1.65	4.4; 2.32	.17
Eosinophils	1–7	1–11	3.6; 1.78	5.9; 2.92	.02*
Neutrophils	0–5	2–9	2.0; 1.63	6.1; 2.42	.00*
Lymphocytes	5–20	6–25	9.8; 4.87	16.7; 7.32	.01*
Monocytes	0–3	0–9	1.0; 1.05	4.5; 3.07	.00*

Abbreviation: *SD*, standard deviation.

*Significant (*p* < .1) difference between the mean cell count in normal and infected tissues.

### 3.2 Leukocyte counts

There was a statistically significant difference between the number of eosinophils, neutrophils, lymphocytes and monocytes (Table 2) between the uninfected and infected tissue. No significant difference was observed for basophil counts (*p*‐value .17).

### 3.3 Infection parameters

Parasite prevalence varied between 11.77% and 100%, mean intensity between 1.5 and 5.7 (Table 3). In a single host, 216 *S. petterae* were recorded, indicating a mass aggregated infection, the remainder of the data also suggested aggregation. Larval infections were only recorded during summer sampling at the Vaal Dam in 2018, all specimens were from a single host. During all other surveys, only adult nematodes were collected.

**TABLE 3:** Summary of the collection data for *Spinitectus petterae* from the Summer of 2018 to the Summer of 2019 from three localities

	All hosts	Infected hosts	Total number of parasites	Location	Prevalence	Intensity range	Mean intensity	Abundance
Summer								
Vaal Dam. Feb, 2018	3	1	8 (larvae)	Rectum (detached)	33.33%	–	–	–
Vaal Dam. Mar, 2019	24	17	96	Stomach	73.91%	1–23	5.7	4.2
Vaal River Barrage. Mar, 2019	17	2	11	Stomach	11.77%	3–8	5.5	0.6
Grootdraai Dam, April, 2019	1	1	216	Stomach	100.0%	–	–	–
Spring								
Grootdraai Dam. Oct, 2018	10	2	3	Stomach	20.0%	1–2	1.5	0.7

## 4 DISCUSSION

*Spinitectus* induced pathology varies greatly depending on the host and parasite species, with *S. micracanthus,* not impacting *L. macrochirus* notably (Keppner, 1975), while *S. carolini* in *L. cyanellus* caused damage to the mucosa and submucosa and caused the formation of ulcers alongside yellow mucus (Meguid & Eure, 1996). *Spinitectus* *petterae* occurred in the stomach, attached to the cardiac and pyloric regions, similar to *S. jamundensis* in *P. lineatus* (Ramallo et al., 2000). However, *S*. *jamundensis* penetrated the muscular layer in the cardiac region, while *S*. *petterae* only penetrated to the submucosa. Specimens of *S. petterae* in the cardiac region were deeply embedded and difficult to remove, while those in the pyloric region detached easily. This may suggest that larvae temporarily attach to the pyloric region and then migrate towards the cardiac region to attach, mature, aggregate and reproduce. This agrees with the observations by Ramallo et al. (2000) who reported only small numbers of larval *Spinitectus* sp. attached to the pyloric region of the stomach of *P. lineatus*. Jilek & Crites (1982a) and Keppner (1975) reported that *S. carolini* and *S. micracanthus* larvae migrate, but these do not cause severe pathology. Other species occurred in the anterior intestine, pyloric cecae and rectum of their hosts, with the 3rd stage larvae occasionally encysting in the mesenteries (Jilek & Crites, 1982a, 1982b), none of which was observed in the present study.

In the present study, both acute and chronic pathology was reported, localized to the attachment site with the surrounding areas not affected. Ulceration, with no infiltration of leukocytes or fibrosis at the ulceration site, was interpreted as acute pathology caused by *S. petterae*. It is speculated that *S. petterae* tunnels through the columnar epithelium, lamina propria and gastric glands. These tissues are mechanically disrupted by the movements and insertion of annular spines into the tissue. The ulcer, which presents as an elongated tunnel, is similar to the ‘migration tracts’ observed by Jilek & Crites (1982a) for *S. carolini*.

Ulcers that occurred adjacent to the granulomas and associated with embedded *S. petterae*, were most likely produced by migrating or not fully attached specimens. Erythrocyte and leukocyte infiltration was absent in the area surrounding ulcerations and could indicate an early‐stage infection (Goodman & Fuller, 2009). With the disruption and destruction of the epithelial layer, lamina propria and gastric gland layer, their function as a protective lining may be impaired. Furthermore, gastric secretions may be similarly impacted with lesions providing an entry point for host gastric secretions or secondary infection by gastrointestinal bacteria (El‐Mansy, 2011).

Attempting to identify the food source of *S. petterae*, specimens were sectioned, but no host tissue was observed inside the vestibule or oesophagus. The fibrous tissue of the granuloma was continuously dislodged, the telescopic cephalic area of *S. petterae* possibly dislodging the granuloma tissue when the annular spines disrupt and tear into it. Liquefaction of these cells occurred and may explain the absence of cellular tissue within the vestibule of adult nematodes and on the lips of larvae. Disrupted tissue close to the body of *S. petterae* showed liquefactive necrosis. Adigun et al. (2021) suggest liquefactive necrosis is caused by bacteria or neutrophils releasing lysosomal enzymes. This cellular material is presumably ingested by *S. petterae* with the aid of the teleostomi and muscular oesophagus creating a vacuum, similar to plant parasitic nematodes (Gaugler & Bilgrami, 2003). Bacteria were not present between the destructed cells but were observed in the vestibule and on the cephalic structures of *S. petterae,* together with leukocytes. These bacteria could be from the *C. gariepinus* microbiome, a part of *S. petterae* microbiome or from the ingested material. Therefore, it is speculated that *S. petterae* either actively feeds on shredded granuloma tissue and/or on bacteria.

Foreign body granuloma formation occurs due to chronic infection and gastric excretion can be reduced by the presence of a granuloma or multiple merged granulomas, influencing digestion and causing malnutrition in the host (Bjørgen et al., 2020). Furthermore, goblet cells undergo hyperplasia, to increase secretions and to form a barrier to separate and eventually eliminate the chronic nematode infection in fish, which is considered an important response to infections (Grencis et al., 2014). This elimination of nematodes is also often associated with the production of yellow mucus (Grencis et al., 2014). However, chronic infection of *S. petterae* in *C. gariepinus* displayed no goblet cell hyperplasia nor thick yellow mucus production, instead the mucus was opaque and white. Similarly, most other *Spinitectus* species do not display goblet cell hyperplasia or yellow mucus (Hoffman, 1975; Jilek & Crites, 1982a; Keppner, 1975; Ramallo et al., 2000), but *S. carolini* from *L. cyanellus* does (Meguid & Eure, 1996).

The average mean intensity of *S. petterae* in the present study was always above five, excluding the 216 individuals recorded from a single host. Higher parasite intensities cause more severe pathology, and infection above five was considered severe in *S. carolini* in *L. cyanellus* (Meguid & Eure, 1996). Similar to other nematodes, a possible aggregation was observed. Infection parameters may also be influenced by seasonality, host sex, host size, sample size and water quality.

In conclusion, the pathology caused by *S. petterae* to the cardiac stomach region of *C. gariepinus* is similar to that of *S. carolini*, described from the anterior intestine of *L. macrochirus* by Jilek and Crites (1982a), and *S. jamundensis* from the cardiac and pyloric stomach regions of *P. lineatus* by Ramallo et al. (2000). Acute ulcerations and chronic granuloma formation affect gastric secretions and ultimately digestion. This may have a serious impact on aquaculture production if the infected intermediate host (aquatic arthropods such as mayflies or crustacea) enters the environment.

## CONFLICT OF INTEREST

The authors declare that they have no competing interests.

## Data Availability

The raw data generated in the study are not included in this publication but are available from the corresponding author on reasonable request.
